# Pamphlets Received

**Published:** 1856-10

**Authors:** 


### *Pamphlets Received.*

*The Mutual Responsibilities of Physicians and the Community. Being an Address to the Graduating Class of the Medical College of the University of Michigan.* By Henry P. Tappan, D. D., LL. D., Chancellor of the University of Michigan.

Dr. Tappan has, in this address, come up to the true standard of legitimacy. We have heard, on what we suppose good authority, that he went to Michigan an avowed homœopathist. If so, we should judge from the sound character of this address, that he has returned to the safe paths.

*Remarks on Vesico-Vaginal Fistula, with an account of a new mode of Suture, and Seven successful operations.* By N. Bozeman, M. D., of Montgomery, Alabama.

Dr. Bozeman's suture is a "button suture," indescribable without plates. Since the publication of this pamphlet he has reported other cases, with remarkable success attending them.

*Report of the Pennsylvania Hospital for the Insane, for the year* 1855. By Thomas S. Kirkbride, M. D., Physician to the Institution.

Dr. Kirkbride makes his usual intelligent and interesting report.

*Essays on the Physiology of the Nervous System, with an Appendix on Hydrophobia.* By Benjamin Haskell, M. D., of Rockport, Mass.

We regret that we have not found time for the perusal of this pamphlet. A glance at its pages indicates that its reading would be profitable, and we accordingly lay it aside for a more convenient season.

*Annual Report of the Commissioners of Emigration, of the State of New York, for the year ending Dec.* 31, 1855. *Including the Medical Report.*

The management of the various mammoth institutions under the control of the commissioners, is a subject in which every citizen has a deep personal interest. Thus far it has been free from those charges of peculation and dishonesty which have attached to so many of our public charities.

*Constitution and By-Laws of the Medical Society of Minnesota. Organized Dec.*, 1855.

As might be expected, our subscribers in St. Pauls are the leaders in this effort to concentrate and give efficiency to the action of the profession in Minnesota. Their by-laws and code of ethics are admirable, and indicate a healthy vigor in the sentiment of our co-laborers in the west. Their Table of Fees is, if anything, a little higher than that recognized in Buffalo. Let our country subscribers, who are toiling their lives away on a practice which costs more than it pays, and makes that man the poorest who has most to do, take notice that in Minnesota they have $1 to $2 for each day visit, $3 to $5 for each night visit, and $1 per mile for traveling, $10 for a consultation, and so on throughout the list.

The practitioners of Minnesota, having set a decent price on themselves, will be estimated accordingly. The new society has our best wishes for a long and prosperous existence.

